# Observational Study of Lipid Profile and C-Reactive Protein after a Seven-Day Fast

**DOI:** 10.3390/nu13010255

**Published:** 2021-01-17

**Authors:** Valeria Galetti, Marica Brnic, Benjamin Lotin, Mauro Frigeri

**Affiliations:** 1 Laboratory of Human Nutrition, Institute of Food, Nutrition and Health, ETH Zurich, 8092 Zurich, Switzerland; vg@vmmt.ch; 2 VMMT Research, 6950 Tesserete, Switzerland; mb@vmmt.ch; 3 Department of Health, Swiss Distance University of Applied Sciences (Fernfachhochschule Schweiz—FFHS), 3900 Brig, Switzerland; 4 Centre Interlude Bien-Être, 1873 Val d’Illiez, Switzerland; benjamin.lotin@mac.com; 5 Fondazione Hospice Ticino, 6900 Lugano, Switzerland

**Keywords:** fasting, periodic fasting, lipid profile, LDL, HDL, triglycerides, CRP, IGF-1, body composition

## Abstract

Fasting is becoming an increasingly popular practice. Nevertheless, its clinical benefits and possible inconveniences remain limitedly evaluated. We observed the effects of a seven-day fast conducted in a non-medical center located in the Swiss Alps. Clinical parameters were measured on the first and last day of fasting (D1 and D7), and two months later (D60). Among the 40 participants, blood analyses were done on 25 persons with an increased metabolic risk, with the primary goal of assessing the lasting effect on low-density lipoprotein (LDL) cholesterol. By comparing D60 with D1, high-density lipoprotein cholesterol (HDL) (+0.15 mmol/L) and insulin-like growth factor-1 (IGF-1) (+2.05 mmol/L) increased (both *p* < 0.009), all other blood parameters (LDL, glucose, total cholesterol, triglycerides, C-reactive protein (CRP)) did not change; weight (−0.97 kg) and hearth rate (−7.31 min^−1^) decreased (both *p* < 0.006). By comparing D7 with D1, total cholesterol (+0.44 mmol/L), triglycerides (+0.37 mmol/L) and CRP (+3.37 mg/L) increased (all *p* < 0.02). The lack of LDL variation at D60 may be due to the low metabolic risk level of the participants. The increase of total cholesterol, triglycerides and CRP at D7 warrants studies to understand whether such fluctuations represent a stress reaction to the fasting state, which may vary in different fasting types.

## 1. Introduction

Fasting is gaining recognition among the scientific community as a practice to prevent diseases that cause most morbidity and mortality in Western populations (i.e., metabolic syndrome, diabetes, cardiovascular disease, cancer, and dementia) [1,2,3,4]. Metabolic changes, which allow for physiological adaptation to the absence of food intake, occur during fasting: activation of lipid catabolism with resulting ketosis [5], reduction in insulin-like growth factor 1 (IGF-1) [6], activation of autophagy [7], and increase in stem cell production [8] are all examples of physiologic changes and processes that have been documented in association with fasting. Combined, these observations suggest that fasting may promote longevity in good health [9].

In animal models, continuous caloric restriction, a diet characterized by a caloric intake that is slightly below the daily requirement, has beneficial effects on longevity similar to those observed with fasting [10]. However, because this low-calorie diet requires important sacrifices and lifestyle changes, it is very challenging to implement on the long term. Moreover, it has been shown to have negative effects on body composition [11,12]. Fasting, which can be repeated regularly but for limited periods of time, does not have such disadvantages.

In Western countries, the most common types of fasting are: (1) time restricted feeding [13] (e.g., 16:8 fasting, in which the daily consumption of food only occurs during eight hours of the day, interrupted by 16 consecutive hours of fasting), (2) intermittent fasting [2,14] (e.g., 5:2 fasting, in which five days of normal food consumption are alternated with two consecutive days of fasting), (3) periodic fasting [15] (fasting for three or more days up to 21 days or, in rare cases, more), and (4) fasting mimicking diet [16] (an advanced, patented form of periodic fasting characterized by a 5-day low-sugar and a low-protein intake). It is not known which approach to fasting provides more physiological benefits and the relative physiological advantages and disadvantages of the various types of fasting are far to be fully understood. One of the disadvantages of periodic fasting, which can last for over three weeks and involves a caloric intake that is well below daily requirements (less than 50%), is the loss of lean body mass.

In the past two decades, many non-medical centers were opened in Europe (e.g., “jeûne et randonnée”, i.e., fasting-and-hiking), offering the experience of undertaking a periodic fast inspired by the Buchinger method. This method, which arose in Germany in the first half of the last century, is also applied in several medical clinics throughout Europe [17]. It is based on an intake of approximately 200–500 kcal per day, made up of abundant liquids, fruit juices, vegetable broths and honey, in conjunction with light-to-moderate physical activity and stress-management activities.

In this article, we present data collected during a prospective observational study in a non-medical fasting center in Switzerland, where the participants fasted for one week and undertook physical activity and stress management (e.g., walking, Pilates, yoga, and painting), under the supervision of naturopaths.

The purpose of our study was to assess the effects of a one-week periodic fast on selected markers of good health. Low-density lipoprotein cholesterol (LDL) has a known role as cardiovascular risk factor and its decrease with non-pharmacological and pharmacological interventions is a goal for good cardiovascular prevention [18]. Our primary objective was to assess whether a one-week periodic fast had a lasting effect on the LDL, assessed before the beginning of the fast and two months after. Our secondary objective was to assess the fasting short-term effect on LDL, measured right after the end of the fast, and its short-term and lasting effect on other blood measurements, clinical parameters and body composition.

## 2. Materials and Methods

The study took place between May 2019 and October 2019 at the Centre Interlude Bien-Être (Val d’Illiez, Switzerland), a non-medical fasting-and-hiking center. In this facility, where the fast is inspired by the Buchinger method, the diet consisted of diluted fruit juices in the morning and vegetable and miso broths in the evening, reaching a maximum total intake of approximately 300 kcal per day. Frequent hydration with water and infusions was recommended. Physical activity was light, rarely moderate. Physical activity and stress management were voluntary. Some participants privately underwent colonic irrigation before starting the fast in a distinct health center.

We recruited the participants by sending an invitation letter to all Swiss people who booked a residential fast at the Centre Interlude Bien-Être. The inclusion criteria were: (1) signature on the informed consent, (2) age between 20 and 70 years, (3) body mass index (BMI) above 18 kg/m^2^, (4) no diagnosis of diabetes mellitus, (5) not taking a hypoglycaemic medication, (6) not known eating disorders (anorexia, bulimia, orthorexia nervosa), (7) not known metabolic diseases where fasting is contraindicated, (8) non-pregnant, and (9) non-lactating.

The study was registered on a primary clinical trial registry with number ISRCTN90206986 and the research protocol was approved by the Cantonal Ethics Commission for Human Research of the Canton of Vaud, Switzerland (BASEC number 2019-00415).

The study followed a prospective observational design. The invitation letter included a first form for the collection of medical information and details of eating habits and physical activity, which was filled out and returned by mail to the investigators along with the signed informed consent. On the first day of fasting (D1), immediately after arrival at the study site, data was collected on weight, height, abdominal circumference, arterial blood pressure (AP), heart rate, body temperature and body composition. This data was also recorded on the last day of fasting (D7), and approximately two months after the beginning of the fast (D60). Shortly before or after D60, the participants received a second form for the collection of medical information, details of eating habits and physical activity, which was filled out and returned by mail to the investigators. We did not provide any advice on dietary or physical activity to be applied between D7 and D60.

The naturopaths present on the study site were trained by the investigators on how to obtain and record clinical parameters. Body weight was measured to the nearest 0.1 kg using a calibrated scale; abdominal circumference was measured horizontally to the nearest 1 cm and at the narrowest point beneath the lowest rib using a metric tape; AP and heart rate were taken in a seated position on the right arm and measured to the nearest 1 mmHg and to the nearest 1 min^−1^, respectively, using an Axapharm A08 blood pressure monitor (Axapharm, Baar, Switzerland); body temperature was measured to the nearest 0.1 °C using a Braun NTF3000 thermometer (Braun GmbH, Kronberg, Germany); body composition was assessed using the multi-frequency bio-impedance analyser Nutriguard MS (Data Input GmbH, Pöcking, Germany) which measures resistance and reactance at 5/50/100 kHz to the nearest 1 Ω. The bio-impedance results were processed by the investigators using the Nutriguard Plus v5.5.x software (Data Input GmbH, Pöcking, Germany), which calculated the fat-free mass (FFM), total body water (TBW), fat mass (FM), body cell mass (BCM), phase angle (PA) and extra-cellular mass (ECM). Body height (rounded to 0.01 m) was sourced from the participant’s identification document.

Three blood samples were taken on D1, D7 and D60 from a subsample of participants (from now on the blood analysis group, BAG) with the following criteria: BMI ≥ 30 kg/m^2^, and/or abdominal circumference ≥80 cm for women and ≥94 cm for men, and/or systolic AP ≥ 130 mmHg, and/or diastolic AP ≥ 85 mmHg, and/or taking an anti-hypertensive medication, but not taking a lipid-lowering medication. Blood was drawn from the ante-cubital vein by trained nurses using two types of tubes (a 2.7-mL fluorinated tube for blood glucose and a 7.5-mL gel tube for serum). The blood was analyzed to determine the fasting glucose, the lipid profile including total cholesterol, high-density lipoprotein cholesterol (HDL), LDL and triglycerides, C-reactive protein (CRP), and insulin-like growth factor-1 (IGF-1).

Blood analysis was carried out through the local medical center (Centre médical de Val d’Illiez, Val d’Illiez, Switzerland) by the local laboratory (Dianalabs Valais, Sion, Switzerland), accredited according to international standard ISO 15189:2012 and Swiss standard SN EN ISO 15189:2013. On D1 (always a Saturday), the blood withdrawals were performed by an independent nurse, blood samples were stored in the medical center at <−18 °C (for IGF-1 analysis) or at <5 °C (for the analysis of all other parameters) and transferred to the laboratory on the following Monday. On D7 (always a Friday), the blood withdrawals were carried out at the medical center and immediately transferred to the laboratory. On D60 (either a Friday or a Saturday), the corresponding procedures were followed. All parameters but IGF-1 were analyzed by means of a modular Roche/Hitachi cobas c501 analyzer (Roche Diagnostics GmbH, Mannheim, Germany) using CFAS calibrators (Calibrator For Automated Systems, Roche Diagnostics GmbH, Mannheim, Germany) according to the following methods: glucose, enzymatic colorimetry with hexokinase (laboratory-specific inter-essay coefficient of variation (CV): 1.1%; limit of detection (LOD): 0.11 mmol/L); total cholesterol and triglycerides, enzymatic colorimetry (CVs: 1.7% and 1.5%; LODs: 0.1 mmol/L and 0.1 mmol/L; respectively); LDL and HDL, homogeneous enzymatic colorimetry (CVs: 1.3% and 1.2%; LODs: 0.1 mmol/L and 0.08 mmol/L; respectively); CRP, immunoturbidimetry (CV: 2.1%; LOD: 0.6 mg/L). IGF-1 was analyzed by electrochemiluminescence immunoassay (ECLIA) (CV: 3.5–7.2%; LOD: 4.4 ng/mL) using an IDS-iSYS multi-discipline automated system (Immunodiagnostic Systems, Tyne & Wear, UK) and World Health Organization international standard for IGF-1 (NIBSC code 02/254).

To assess the size of the BAG subsample, we calculated 18 participants were needed to detect a LDL difference between D1 and D60 (our primary objective) of 1.1 mmol/L in two paired groups, with a standard deviation (SD) of 1.5 mmol/L, a type-1 error of 5%, and a power of 80%. We estimated at 20% the prevalence of participants eligible for the BAG [19], thus bringing to 100 the total number of participants.

### Statistical Analysis

We conducted data analyses using R statistical programming environment (v3.6.3, R Core Team 2020, R Foundation for Statistical Computing, Vienna, Austria) with packages nlme [20] and lsmeans [21]. We assessed data normality by using the Shapiro-Wilk W-test. When departing significantly from normality (W < 0.97), we applied logarithmic and square root transformations, and chose the transformation that scored highest W. We investigated the effect of time (D1, D7 and D60 as fixed factor) and the effect of subject (as random factor) on all measured parameters (anthropometric and body composition data, abdominal circumference, body temperature, heart rate, AP, circulating levels of fasting glucose, total cholesterol, HDL, LDL, triglycerides, CRP and IGF-1) by using mixed effect model analysis. We carefully evaluated all models for heteroscedasticity by visual inspection of the models’ residuals on the Tukey-Anscombe plot. When the effect of time was significant, we performed a post-hoc analysis to investigate the difference between time points (D60–D1, D7–D1, D60–D7). Post-hoc analysis is presented as mean difference and *p*-value. In addition, we evaluated the association of the change before and after the fasting week (D7 minus D1) between CRP, total cholesterol and triglycerides by performing the Spearman’s rank correlation test. Spearman’s rank correlation test is presented as coefficient rho and *p*-value. Significance level was set to <0.05.

## 3. Results

### 3.1. Participants

Forty-one people signed the informed consent form, and all were eligible to participate in the study. One participant did not submit the first form and immediately left the study voluntarily: the data for D1 include 40 participants. One participant left the trial voluntarily before the end of the first week of fasting: the data for D7 include 39 participants. Three participants left the trial voluntarily and did not undergo the measurements on D60: the data for D60 include 36 participants.

Among the 41 recruited participants, 29 were eligible for the BAG. However, four participants did not provide a blood sample due to organizational problems. There were 25 participants with blood analysis, with one participant refusing blood sampling on D60. Thus, the available data include 25 participants at D1 and D7, and 24 participants at D60.

Table 1 shows the results of the BAG subgroup blood markers. Table 2 shows the body and body composition measurements for the entire group, once the needed number of participants for the assessment of the primary objective (as in accordance with the sample size calculation) had been obtained.

### 3.2. BAG and Blood Analyses

Of the 25 participants in the BAG, 22 were women, and the mean (±SD) age was 51.36 (±7.86) years. About 4% of the participants satisfied all five inclusion criteria (BMI ≥ 30 kg/m^2^, and/or abdominal circumference ≥80 cm in women and ≥94 cm in men, and/or systolic AP ≥ 130 mmHg, and/or diastolic AP ≥ 85 mmHg, and/or taking an antihypertensive medication), 12% satisfied four of them, 12% satisfied three, 32% satisfied two and 40% satisfied one criterion. The most observed inclusion criterion in the 25 participants of the BAG group was a high abdominal circumference, in 96% of the participants. A high systolic AP, high BMI, high diastolic AP and the intake of anti-hypertensive medication were found in 52%, 28%, 20% and 8% of cases respectively. None of the participants were taking a lipid-lowering medication. On D1, mean (±SD) BMI was 26.93 (±4.20) kg/m^2^, abdominal circumference was 95.00 (±11.44) cm, systolic AP was 130.04 (±16.24) mmHg, and diastolic AP was 79.56 (±7.29) mmHg (Supplementary Table S1). Mean (±SD) fasting glucose was 4.50 (±0.33) mmol/L, total cholesterol was 5.31 (±0.89) mmol/L, LDL was 3.62 (±0.97) mmol/L, HDL was 1.60 (±0.34) mmol/L, triglycerides was 0.96 (±0.30) mmol/L, IGF-1 was 14.18 (±6.79) mmol/L and CRP was 1.79 (±2.26) mg/L; the mean values of fasting glucose, HDL, triglycerides, IGF-1 and CRP were within the normal range provided by the laboratory, whereas the mean values of total cholesterol and LDL were high (Table 1). None of the BAG participants had a metabolic syndrome according to criteria by the International Diabetes Federation [22].

When we compared the values on D7 with D1, we observed a significant decrease in mean (95% CI) fasting glucose (−0.83 (−1.12, −0.54) mmol/L, *p* < 0.001), HDL (−0.12 (−0.18, −0.05) mmol/L, *p* = 0.003), IGF-1 (−4.90 (−6.60, −3.20) mmol/L, *p* < 0.001); a significant increase in mean total cholesterol (0.44 (0.22, 0.66) mmol/L, *p* = 0.018), triglycerides (0.37 (0.19, 0.54) mmol/L, *p* < 0.001), CRP (3.37 (1.92, 4.82) mg/L, *p* < 0.001); no significant changes in LDL. On D60, compared with the values on D1, we observed a significant increase in mean (95% CI) HDL (0.15 (0.09, 0.20) mmol/L, *p* < 0.001) and IGF−1 (2.05 (0.34, 3.75) mmol/L, *p* = 0.008); no significant changes in fasting glucose, total cholesterol, LDL, triglycerides or CRP.

The null hypothesis of the primary objective was therefore verified: no significant changes in LDL were detected two months after the start of the fast.

### 3.3. Clinical Parameters

In the entire group comprising 40 participants, 35 were women, and the mean (±SD) age was 50.03 (±9.48) years. On D1, the mean (±SD) weight was 73.45 (±13.87) kg and the mean (±SD) BMI was 25.55 (±4.04) kg/m^2^, the study population was therefore slightly overweight. The mean (±SD) systolic and diastolic APs were 124.93 (±17.45) mmHg and 77.80 (±8.91) mmHg, respectively, average values for a normotensive population. The mean (±SD) abdominal circumference was 88.70 (±13.46) cm, with values for four men (80%) and 23 women (68%) above the limit specified as one of the diagnostic criteria of metabolic syndrome according to the International Diabetes Federation [22]. The mean (±SD) heart rate was 74.03 (±9.62) min−1, indicating a normocardial population. The mean body temperature (±SD) was 36.84 (±0.60) °C (Table 2).

When we compared clinical parameters taken on D7 with those at D1, we observed a significant decrease in mean (95% CI) weight (−3.70 (−4.12, −3.28) kg, *p* < 0.001), BMI (−1.30 (−1.45, −1.16) kg/m^2^, *p* < 0.001), abdominal circumference (−4.47 (−5.39, −3.56) cm, *p* < 0.001), systolic AP (−6.36 (−10.84, −1.87) mmHg, *p* = 0.013), body temperature (−0.44 (−0.61, −0.26) °C, *p* < 0.001); no significant changes in diastolic AP and heart rate. On D60, compared with the values on D1, we observed a significant decrease in mean (95% CI) weight (−0.97 (−1.72, −0.21) kg, *p* = 0.005), BMI (−0.35 (−0.61, −0.09) kg/m^2^, *p* = 0.005), heart rate (−7.31 (−10.66, −3.95) min^−1^, *p* < 0.001); no significant changes in abdominal circumference, AP or body temperature.

### 3.4. Body Composition

The mean (±SD) body composition values on D1 were (Table 2): FFM 49.48 (±7.68) kg, TBW 36.22 (±5.63) kg, FM 24.15 (±8.66) kg, BCM 26.06 (±5.13) kg, PA 6.14 (±0.63), and ECM 23.41 ± 3.00 kg. On D7, compared with D1, we observed a significant decrease in mean (95% CI) FFM (−3.84 (−4.32, −3.35) kg, *p* < 0.001), TBW (−2.81 (−3.16, −2.46) kg, *p* < 0.001), BCM (−1.56 (−1.83, −1.29) kg, *p* < 0.001), ECM (−2.28 (−2.63, −1.93) kg, *p* < 0.001); a significant increase in mean (95% CI) PA (0.23 (0.13, 0.33), *p* < 0.001); no significant change in FM. On D60, compared with D1, we observed a significant decrease in mean (95% CI) FM (−2.16 (−2.93, −1.39) kg, *p* < 0.001), PA (−0.41 (−0.52, −0.29), *p* < 0.001), BCM (−0.59 (−1.05, −0.13) kg, *p* < 0.001); a significant increase in mean (95% CI) FFM (0.84 (0.28, 1.39) kg, *p* < 0.046), ECM (1.41 (1.06, 1.76) kg, *p* < 0.001); no significant change in TBW.

## 4. Discussion

In this article, we present the data collected until we reached the number of participants in the BAG required to assess the primary objective (the difference in LDL concentration between D1 and D60), as established in the study protocol. We do not present the data on eating habits and physical activity collected using the questionnaires. In this prospective observational trial, we show clinical parameters and blood analysis carried out right before fasting started, on completion of a seven-day fast and two months after the start of the fast, in participants spending one week at a non-medical fasting center and practicing a seven-day fast inspired by the Buchinger method.

Two previous observational trials, carried out in medical settings, monitored similar parameters at the end of a one-week fast: the first observed a population of obese women [23], whereas the second observed a population of participants staying at a clinic for at least 10 days [24]. In another trial, this time with a control group, similar parameters are described at four months after starting a one-week fast in a population suffering from type-2 diabetes [25]. Finally, there is one observational study performed in a population with metabolic syndrome and/or type 2 diabetes that only described BMI and glucose metabolism at the end of a fast lasting 7–18 days and 80 days after the start of fasting [26].

Our primary objective, LDL, did not change after fasting, and is in line with the results obtained from two other trials: the aforementioned trial in the population with type-2 diabetes [25], and a randomized controlled trial in healthy volunteers who participated in a fasting mimicking diet trial [16]. We chose to measure LDL two months after fasting as our primary objective given the known role of LDL as a cardiovascular risk factor [18]. In order to calculate the required sample size, we chose to use an effect size of 1.1 mmol/L, which refers to the decrease in LDL observed when patients undergo a treatment deemed effective [27].

In clinical practice, primary prevention treatment with statins may be prescribed to patients with an LDL value above 4.9 mmol/L [28]. Three participants had an LDL above this threshold. Their baseline mean (±SD) LDL was 5.27 (±0.12) mmol/L and decreased to 4.37 (±0.40) mmol/L at D60 (mean reduction of 17%), thus below the threshold. These three cases might lead us to suspect that for a group with high-risk LDL values (>4.9 mmol/L), fasting may be a therapeutic alternative in primary cardiovascular prevention, a fact that deserves to be confirmed in randomized controlled trials in at-risk patients. Similar results were obtained in a post hoc analysis of participants with high LDL in a trial involving a fasting mimicking diet [16].

There were no significant changes in CRP, total cholesterol and triglycerides at D60. At D7, instead, these three values significantly increased, and this change was partially unexpected. With regards to CRP, at D7, mean CRP increased by 3.4 mg/L. Although this phenomenon has been observed previously in other fasting trials [23,24,29], a satisfactory explanation for this increase is still lacking. While we agree that a CRP increase is the response to the activation of the acute phase [30] following the activation of the hypothalamic-pituitary-adrenal axis [29], we nevertheless wonder to what extent a CRP increase can be a marker of the activation of the metabolic changes specific to fasting. Moreover, the link between the known anti-inflammatory effect of fasting [3] and this apparent “slight” inflammatory activation during fasting is still not clear. Eleven participants had a moderate-to-high cardiovascular risk at D1 with a CRP ≥ 1 mg/L [31]. Among these, from D1 to D60, the mean CRP decreased from 3.46 (±2.59) mg/L to 2.61 (±1.25) mg/L.

With regards to triglycerides and total cholesterol, at D7, mean triglycerides and total cholesterol increased both by 0.4 mmol/L. A concomitant increase in these values was observed previously in a trial studying the effect of a fasting mimicking diet in oncology patients [32]. The triglycerides increase can be explained by the activation of the acute phase response [33], but this was not observed in those studies reporting an increase in CRP [23,24]. The total cholesterol increase is a phenomenon that cannot be explained by activation of the acute phase response. In fact, although an increase in very LDL and small dense LDL has been reported during cases of septicemia [34], total cholesterol typically decreases in these situations [33]. On the other hand, an increase in total cholesterol has been described in less physiologically stressful situations than septicemia, for example when taking written exams [35]. Fasting could be a kind of stress that leads to a transient increase in total cholesterol, an event previously described in the context of a 24-h water fast [36] in which triglycerides decrease, and a 10-day water fast [37] in which triglycerides were not reported.

We investigated the correlations between the D1-to-D7 changes in triglycerides, total cholesterol and CRP. There was a slight positive correlation between total cholesterol and triglycerides (rho = 0.62, *p* < 0.001). There was no correlation between total cholesterol and CRP (rho = −0.31, *p* = 0.127) and between triglycerides and CRP (rho = −0.098, *p* = 0.641). The concomitant increase in triglycerides, total cholesterol and CRP could reflect metabolic changes specific to an extended fast in which, for example, hydration is compromised. In a study investigating the physiologic effects of a dry fast, an increase in mean CRP of 4.9 mg/L was observed [29], but the lipid profile was not described. A dry fast would intuitively enhance the activation of the acute phase response compared with the fasting practices inspired by Buchinger. According to the body composition data, we observed a significant decrease in mean body water fraction, from 49.8% on D1 to 48.2% on D7. This decrease, albeit small, occurs systematically and led us to suspect a tendency to dehydrate during fasting among our cohort.

The pattern of the change of triglycerides and total cholesterol that we observed in our study is an atypical feature that made us reflect on the diet consumed in the fasting center that hosted the present study. We think that the consumption of a miso soup (produced from the fermentation of soya typical of Japanese cuisine) deviates significantly from the traditional Buchinger method [15]. It is known that soybean fermentation products have biological properties on fat metabolism [38], but we do not believe that the ingestion of miso can explain alone the progression of triglycerides and total cholesterol in this cohort.

The reduction in AP is a known effect of fasting in people with hypertension [39]. Our cohort had a mean (±SD) AP of 124.93/77.80 (±7.45/8.91) mmHg. The lack of decrease in diastolic AP can be partly explained by the APs in the normal range for almost all participants at baseline. The nine participants with a systolic AP ≥ 140 and/or diastolic AP ≥ 90 mmHg on D1, had an average AP of 147.00/86.56 (±9.46/8.92) mmHg. Among these, no changes in mean diastolic AP were observed at D7 and D60, but the mean change in systolic AP is evidently higher in these nine participants than in the whole cohort: −21.00 mmHg (versus −6.36 mmHg) and −15.29 mmHg (versus −4.08 mmHg) for D7 and D60, respectively.

On D60, we observe that the decrease in body weight and BMI are maintained when compared with D1 (−0.97 kg and −0.35 kg/m^2^, respectively). The increasing trend observed between D7 and D60 led us to suspect that body weight is regained in the medium to long term. Weight loss maintenance is one of the hard goals in the quest for preservation of good health [40,41]. Fasting techniques involving time restricted feeding and intermittent fasting have shown to produce an effect on weight loss and weight loss maintenance comparable to continuous caloric restriction [42]. Moreover, intermittent fasting is likely to have fewer adverse effects on body composition compared to continuous caloric restriction [11]. At D60, in our cohort, we observed a significant increase in FFM and a decrease in FM, confirmed by the consequent significant increase in the FFM/FM ratio (from 2.19 to 2.50, from D1 to D60). Those changes not only confirm the safety of fasting, but when considered alongside the partial maintenance of weight loss, it provides evidence for a possible improvement in body composition. The same effect has already been observed in a similar context [16]. The role played by periodic fasting in weight loss and the maintenance of weight loss is still not clear. The results of a randomized controlled trial showed that even 80 days after a one-week of fasting providing 300 kcal/day by liquids only, the experimental group maintained a weight loss of 3.5 kg versus 2.0 kg in the control group [25].

We chose the inclusion criteria for the BAG with the intention of creating an at-risk cohort, as in accordance with the literature [16,23], that would benefit more from the fasting program. The BAG inclusion criteria included BMI, abdominal circumference and AP, which we chose from the most readily obtainable, established clinical measures used to diagnose a metabolic syndrome [43]. Over 70% of all included participants were eligible for the BAG. This prevalence was three-to-four times the anticipated 20% that we estimated from prevalence of obesity and hypertension in Switzerland in 2012 [19]. The BAG AP limits (>130 mmHg systolic, >85 mmHg diastolic) that we applied are not those used by the Swiss Federal Statistical Office (>140 mmHg systolic, >90 mmHg diastolic) to define hypertension, thus our anticipated prevalence was too low.

We considered the decrease of IGF-1 and fasting glucose measured on D7 to be proof of the state of fasting. Similar results were also observed in other fasting settings [16,24]. The fact that we did not measure ketone bodies might be deemed a limitation of the study, but we considered the voluntary choice and payment for the stay of the participants as a guarantee of fasting adherence. The observation of the group confirms its validity, even if some participants might have eaten in secret.

To conclude, LDL did not decrease two months after the start of the fast, but there are four further aspects—three of the D60 results and one of the D7 results—that deserve to be highlighted and warrants further research: (1) the maintenance, albeit partial, of weight loss and decreased BMI, accompanied by a possible improvement of body composition, fosters the possible role of periodic fasting in the management of weight loss; (2) the decrease in heart rate, which may reflect an increase in parasympathetic tone [44], is preserved in the medium term; (3) the increase in HDL, which may be an indication of the medium term cardiovascular preventive properties of fasting, is a result corroborated by already suggested trends [16,25]; (4) the behavior of the lipid profile and CRP during fasting has no definitive explication and seems vary in different types of fasting.

This study has some limitations. One limitation was the lack of a control group, which is a characteristic of observational studies. One limitation of the observational study design is that multiple possible confounding factors were not determined in advance. One evident possible confounding factor is linked to the metabolic syndrome and its cardiovascular risk. The selection criteria applied to the group allocated to the collection of data for the primary objective (BAG) were probably set too generously. This had the consequence that the BAG participants had fewer risk factors than we expected. We suspect that the absence of a difference in LDL two months after the start of the fast depends on the actual low risk level of the selected BAG participants. Another limitation was the absence of direct medical supervision in taking clinical parameters, which were carried out by people with training in naturopathy, who do not formally have such competence. According to our checks, however, the naturopaths respected the directions given by the medical study team during a training before the study started. Finally, because the forms in which the participants reported details on eating habits and physical activity have not yet been analyzed, we cannot conclude in which proportion the observed medium-term changes in clinical parameters are due to the fasting week itself vs. possible habits modification engendered by the fasting experience.

We consider important to observe these emerging realities of non-medicalized fasting experiences from a medical point of view, in order to establish their role in health and prevention. This work offers useful elements to improve interdisciplinary collaborations (e.g., between traditional Western medicine and naturopathy) and hopes to make a contribution to the understanding of the complexity of preventive interventions that include elements of physical activity, stress management, good nutrition, and finally also fasting.

## Figures and Tables

**Table 1:** Circulating levels of glucose, cholesterol and triglycerides, C-reactive protein and IGF-1 assessed just before the fasting week started (Day 1), at fasting week completion (Day 7) and at a 2-month follow-up (Day 60) in *n* = 25 generally healthy adults with at least one clinical criterion for metabolic syndrome.

Parameter	Before Fasting Week (Day 1)	After Fasting Week (Day 7)	Follow-Up (Day 60)	*p* for Overall Time Effect
*n*	25	25	24	
Glucose (mmol/L)	4.50 ± 0.33 ^a^	3.67 ± 0.78 ^b^	4.73 ± 0.44 ^a^	<0.001
Total cholesterol (mmol/L)	5.31 ± 0.89 ^a^	5.75 ± 1.21 ^b^	5.54 ± 0.73 ^a,b^	0.024
LDL cholesterol (mmol/L)	3.62 ± 0.97	3.93 ± 1.21	3.76 ± 0.79	0.145
HDL cholesterol (mmol/L)	1.60 ± 0.34 ^a^	1.48 ± 0.35 ^b^	1.74 ± 0.36 ^c^	<0.001
Triglycerides (mmol/L)	0.96 ± 0.30 ^a^	1.33 ± 0.38 ^b^	1.02 ± 0.36 ^a^	<0.001
C-reactive protein (mg/L)	1.79 ± 2.26 ^a^	5.16 ± 4.73 ^b^	1.77 ± 1.38 ^a^	<0.001
IGF-1 (mmol/L)	14.18 ± 6.79 ^a^	9.28 ± 3.70 ^b^	16.51 ± 5.36 ^c^	<0.001

Data are mean ± standard deviation (SD). Data was analyzed using mixed effects models, with time as a fixed factor and subject as a random factor. Post-hoc analysis with Tukey correction was performed to investigate differences between time points. Normally distributed data was analyzed using raw data. Not normally distributed data was analyzed using natural logarithm- or square root-transformed data. ^a, b, c^ Different superscript letters in the same raw indicate significant difference between values at *p* < 0.05 level. HDL, high-density lipoprotein; IGF-1, insulin-like growth factor-1; LDL, low-density lipoprotein.

**Table 2:** Anthropometric parameters, clinical parameters and body composition at fasting week completion (Day 7) and at a 2-month follow-up (Day 60) in *n* = 40 generally healthy adults.

Parameter	Before Fasting Week (Day 1)	After Fasting Week (Day 7)	Follow-Up (Day 60)	*p* for Overall Time Effect
*n*	40	39	36	
% males	12.50			
Age (y)	50.03 ± 9.48			
Height (cm)	169.23 ± 7.28			
Weight (kg)	73.45 ± 13.87 ^a^	69.67 ± 13.84 ^b^	72.52 ± 14.69 ^c^	<0.001
Body mass index (kg/m^2^)	25.55 ± 4.04 ^a^	24.18 ± 3.98 ^b^	25.14 ± 4.39 ^c^	<0.001
Abdominal circumference (cm)	88.70 ± 13.46 ^a^	84.19 ± 12.82 ^b^	87.36 ± 12.81 ^a^	<0.001
Temperature (°C)	36.84 ± 0.60 ^a^	36.39 ± 0.66 ^b^	36.75 ± 0.49 ^a^	<0.001
Cardiac frequency (min^−1^)	74.03 ± 9.62 ^a^	75.46 ± 12.47 ^a^	67.00 ± 10.44 ^b^	<0.001
Systolic blood pressure (mmHg)	124.93 ± 17.45 ^a^	118.51 ± 13.15 ^b^	120.56 ± 14.41 ^a,b^	0.016
Diastolic blood pressure (mmHg)	77.80 ± 8.91	77.74 ± 8.88	76.92 ± 10.10	0.877
Fat-free mass (kg)	49.48 ± 7.68 ^a^	45.40 ± 7.36 ^b^	50.06 ± 7.30 ^c^	<0.001
Fat-free mass (%)	67.98 ± 6.43 ^a^	65.87 ± 5.92 ^b^	70.02 ± 6.58 ^c^	<0.001
Total body water (kg)	36.22 ± 5.63 ^a^	33.23 ± 5.39 ^b^	36.63 ± 5.35 ^a^	<0.001
Total body water (%)	49.76 ± 4.70 ^a^	48.22 ± 4.33 ^b^	51.24 ± 4.82 ^c^	<0.001
Fat mass (kg)	24.15 ± 8.66 ^a^	24.26 ± 8.31 ^a^	22.46 ± 8.92 ^b^	<0.001
Corrected fat mass (kg)	25.57 ± 8.64 ^a^	22.43 ± 8.27 ^b^	24.90 ± 9.13 ^c^	<0.001
Fat mass (%)	32.04 ± 6.45 ^a^	34.10 ± 5.92 ^b^	29.98 ± 6.59 ^c^	<0.001
Body cell mass (kg)	26.06 ± 5.13 ^a^	24.35 ± 4.84 ^b^	25.30 ± 4.30 ^c^	<0.001
Body cell mass (%)	35.66 ± 4.07	35.23 ± 3.97	35.37 ± 3.96	0.744
Phase angle	6.14 ± 0.63 ^a^	6.35 ± 0.67 ^b^	5.72 ± 0.49 ^c^	<0.001
BCM/FFM (%)	52.44 ± 2.91 ^a^	53.44 ± 2.94 ^b^	50.47 ± 2.46 ^c^	<0.001
Extra-cellular mass (kg)	23.41 ± 3.00 ^a^	21.05 ± 2.97 ^b^	24.74 ± 3.43 ^c^	<0.001
Extra-cellular mass (%)	32.31 ± 3.51 ^a^	30.64 ± 3.10 ^b^	34.63 ± 3.46 ^c^	<0.001
ECM/BCM index	0.91 ± 0.10 ^a^	0.88 ± 0.10 ^b^	0.99 ± 0.10 ^c^	<0.001
FFM/FM index	2.19 ± 0.63 ^a^	2.02 ± 0.53 ^b^	2.50 ± 0.79 ^c^	<0.001
Drink volume (L)	1.11 ± 0.50 ^a^	0.52 ± 0.31 ^b^	0.69 ± 0.44 ^b^	<0.001

Data are mean ± SD. Data was analyzed using mixed effects models, with time as a fixed factor and subject as a random factor. Post-hoc analysis with Tukey correction was performed to investigate differences between time points. Normally distributed data was analyzed using raw data. Not normally distributed data was analyzed using natural logarithm- or square root-transformed data. ^a, b, c^ Different superscript letters in the same raw indicate significant difference between values at *p* < 0.05 level. BCM, Body cell mass; ECM, Extra-cellular mass; FFM, Fat-free mass; FM, Fat mass.

## Data Availability

The data presented in this study are available on request from the corresponding author. The data are not publicly available due to ethical restrictions.

## Supplementary Materials

The following are available online at https://www.mdpi.com/2072-6643/13/1/255/s1. Table S1: Anthropometric parameters, clinical parameters and body composition data assessed using Nutriguard plus software just before the fasting week started (Day 1), at fasting week completion (Day 7) and at a 2-month follow-up (Day 60) in *n* = 25 generally healthy adults with at least one clinical criterion for metabolic syndrome.
