# Supplementary material for: Observational Study of Lipid Profile and C-Reactive Protein after a Seven-Day Fast
Source: Nutrients. 2021 Jan 17;13(1):255. doi: 10.3390/nu13010255 (PMC7830333; doi:10.3390/nu13010255)
Supplement: Supplementary file 1 [file nutrients-13-00255-s001.pdf]

Supplementary Table S1. Anthropometric parameters, clinical parameters and body composition data assessed using Nutriguard plus software just before the fasting week started (Day 1), at fasting week completion (Day 7) and at a 2-month follow-up (Day 60) in *n*=25 generally healthy adults with at least one clinical criteria for metabolic syndrome.

**Parameter**	**Before Fasting Week (Day 1)**	**After Fasting Week (Day 7)**	**Follow-Up (Day 60)**
n	25	25	24
% males	12		
Age (y)	51.36 ± 7.86		
Height (cm)	171.00 ± 7.00		
Weight (kg)	79.28 ± 13.86	75.02 ± 13.37	78.80 ± 13.98
Body mass index (kg/m^2)	26.93 ± 4.20	25.52 ± 4.13	26.73 ± 4.53
Abdominal circumference (cm)	95.00 ± 11.44	90.18 ± 11.17	92.83 ± 11.88
Temperature (°C)	36.83 ± 0.61	36.40 ± 0.70	36.75 ± 0.49
Cardiac frequency (min^{-1})	73.40 ± 9.96	76.60 ± 13.83	68.42 ± 11.32
Systolic blood pressure (mmHg)	130.04 ± 16.24	122.24 ± 12.01	123.88 ± 13.89
Diastolic blood pressure (mmHg)	79.56 ± 7.29	80.56 ± 8.94	80.75 ± 8.72
Fat-free mass (kg)	51.84 ± 7.26	47.86 ± 6.71	52.91 ± 7.22
Fat-free mass (%)	66.13 ± 6.50	64.57 ± 6.67	67.94 ± 6.59
Total body water (kg)	37.96 ± 5.32	35.04 ± 4.91	38.72 ± 5.28
Total body water (%)	48.42 ± 4.76	47.27 ± 4.89	49.72 ± 4.83
Fat mass (kg)	27.46 ± 8.95	27.15 ± 8.91	25.89 ± 8.89
Corrected fat mass (kg)	29.04 ± 8.92	25.41 ± 8.69	28.30 ± 9.35
Fat mass (%)	33.90 ± 6.53	35.41 ± 6.66	32.05 ± 6.60
Body cell mass (kg)	27.43 ± 4.94	25.70 ± 4.55	26.74 ± 4.53
Body cell mass (%)	34.90 ± 4.24	34.61 ± 4.39	34.31 ± 4.30
Phase angle	6.18 ± 0.59	6.37 ± 0.62	5.71 ± 0.53
BCM/FFM (%)	52.72 ± 2.67	53.53 ± 2.69	50.41 ± 2.66
Extra-cellular mass (kg)	24.43 ± 2.79	22.15 ± 2.62	26.14 ± 3.20
Extra-cellular mass (%)	31.25 ± 3.16	29.95 ± 3.09	33.60 ± 3.17
ECM/BCM index	0.90 ± 0.09	0.87 ± 0.09	0.99 ± 0.10
FFM/FM index	2.07 ± 0.66	1.93 ± 0.59	2.26 ± 0.70
Drink volume (L)	1.10 ± 0.55	0.51 ± 0.38	0.56 ± 0.38

Data are mean ± SD. BCM, Body cell mass; ECM, Extra-cellular mass; FFM, Fat-free mass; FM, Fat mass.
